# Prospective study relating genotype profiles with race performance in racing pigeons

**DOI:** 10.1007/s13353-022-00697-w

**Published:** 2022-05-04

**Authors:** Geert Kolvenbag, Mark Scott, Arne de Kloet, Ed de Kloet

**Affiliations:** 1 Kennett Square, PA USA; 2 Sarasota, FL USA; 3 Animal Genetics Inc, Tallahassee, FL USA

**Keywords:** Pigeon racing, Lactate dehydrogenase, Dopamine receptor, Feather keratin

## Abstract

This is a prospective study to investigate the impact of genotype profiles on race performance in racing pigeons. Genotypes studied included lactate dehydrogenase A (*LDHA*), dopamine receptor (*DRD*), myostatin (*MSTN*), and feather keratin (*F-KER*), as well as demographic factors such as gender, color, and the mtDNA. This study shows differences within genotypes *DRD456* (*P* = 0.027) and *F-KER* (*P* = 0.018). For *DRD456*, race coefficients were lower (= better performance) for genotype *CT*. For *F-KER*, race coefficients were lower for *GG*, overall, while within the *F-KER TT* genotype race performance was best at longer distances. After including Queen L mtDNA in the model, both the effects of *F-KER* and *DRD456* remained significant. The effect of Queen L mtDNA alone was significant (*P* = 0.004) and mainly driven by the effect in short distance races. In addition, birds with the checker color check had a lower race coefficient than birds with the color blue bar (*P* = 0.0012). Also, this effect was independently significant and remained significant in the multivariate analysis. No differences in race coefficients were seen between genotypes for *LDHA* and *MSTN* nor for the demographic factor of gender. While individual factors were related to differences in race performance, and although one could be tempted to favor a bird with *DRD4 CCCT*–*F-KER TT*–*LDHA AB*–checker color for long distance races, further and larger prospective studies including birds unrelated to our family of birds will be needed to confirm our findings and to determine a superior profile including multiple genetic factors.

## Introduction

For many years, pigeon fanciers have been looking for objective factors related to race performance. With the technical advance of DNA genotyping, several studies have made suggestions for such linkage for one or more genes (Proskura et al. 2015b; Proskura et al. 2017; Dybus et al. 2018). The genotypes explored in this study have been suggested as possibly related to race performance in racing pigeons (*LDHA*, *DRD4*, *MSTN*, *F-KER*, mtDNA) and make biologically sense to impact the performance physiology, while factors such as gender and color are considered not related to race performance.

The *LDHA* gene contains the code for a protein called lactate dehydrogenase A, which is a lactate dehydrogenase enzyme (Farhana & Lappin 2021). Lactate is made by muscle fibers and is what causes the pain in skeletal muscle tissue when doing physical efforts, related to endurance, speed, and power. Skeletal muscles are those muscles involved in movement. It is these muscles that are being trained when an athlete goes from aerobic to anaerobic phase.

During high-intensity physical activity, skeletal muscles need increased amounts of energy. Anaerobic exercise occurs when the body’s oxygen intake is not sufficient for the amount of energy required. To create additional energy, glucose stored in the body as glycogen gets broken down. During the breakdown of glycogen, the lactate dehydrogenase enzyme converts the molecule pyruvate into a similar molecule called lactate, which can be used by the body for energy.

The pain in these muscles during and after high physical exertion is in part a result of lactate buildup. The more an athlete reaches their maximum effort, the more lactate is made by the muscle fibers.

Previous studies have shown that mutations *LDHA* (g.2582481G > A) have been linked to overall performance in racing pigeons (Proskura et al. 2014, 2015a, b). These alleles have been labeled the names A (A) and B (G). Research suggests that genotype A was found significantly more often in top performance pigeons. There are 3 possible genotypes with these alleles, namely BB, AB, and AA. The A allele is a rare variation, found in less than 12% of normal racing pigeons, and less than 1% of pigeons had the statistically more favorable AA genotype (Dybus et al. 2006). It is important to mention that some top racing pigeons are BB genotype, meaning that the A allele is not absolutely necessary to be able to perform at a high level. This suggests that additional genes are involved in race performance including speed and distance. **LDHA hypothesis **the pigeon that is homozygous for A (carries two copies of the A allele) is a better race performance bird.

A relationship between the dopamine receptor D4 (*DRD4*) gene and race performance in pigeons was first reported in 2015 (Proskura et al. 2015b).

In this study, 1380 racing records from 8 (< 400 km) and 6 (> 500 km) long races were taken into account. The study found a number of different variations in the dopamine receptor type 4 gene, of which two were relevant for racing performance. The variances in *DRD4* were found to influence performance at all distances, but only for speed and middle distance races the differences were statistically significant.

Using SNP g.129954C > T *DRD4*a and SNP g.129456C > T *DRD4*b, the variant *CCCC* (combined genotype of both loci) was associated with the lowest overall mean in racing performances. The variants *CTCC* and *CCCT* had higher statistically significant averages; and the *CTCT* variant had a very high mean in racing performance. This difference was statistically significant for on speed and middle distance (< 400 km). In a longer distance (> 500 km), the mean was higher but this was not statistically different in this study. **Hypothesis DRD4a and b** the hypothesis for long distance races is as follows: *DRD4 CTCT* > *CTCC* or *CCCT* > *CCCC*, *CCTT*, *TTCC*, *TTTT* (> is better performance).

*F-KER* mutation of interest is the g.710 T > G in the keratin gene, which results in a cysteine to glycine amino acid change at position 83 (Cys83Gly) in feather keratin, and may be related to racing pigeon racing performance.

The pigeons with the *F-KER TT* genotype had the highest mean of ace points in the long races and in all races overall, while the birds with *GT* scored the best in the short races. Nevertheless, the effect of the polymorphism was significant only in the long races (*P* = 0.0451), in which the pigeons carrying the *TT* genotype showed better racing performance in comparison with those carrying the *GG* genotype (*P* ≤ 0.05) (Proskura et al. 2017).

The loss of the cysteine at position 83 of pigeon feather keratin may affect the structure of feathers with as a result changing their biomechanical characteristics, and thereby may impact the race performance. The **hypothesis for *F-KER*** in long distance races is as follows: *TT* > *TG* or *GG* (> is better performance).

The molecular basis of the *MSTN* mutation of the analyzed SNP is g.11440232C > T; a C → T transition, located in the 3rd exon of the *MSTN* gene in the 287th codon (ACC to ACT) for threonine. It is a silent mutation. One study showed mutation T was more common in higher muscle mass pigeons (Dybus et al. 2013, 2018). The **MSTN hypothesis** is that pigeons with great muscle mass will perform better in longer distance races.

## Methods

In 2019 at one breeding loft, 17 breeding pairs produced 124 young birds; 2 did not have any genetic testing performed. Of the 122 birds with genetic testing information available, 107 were entered in one of 14 one loft races (OLRs) and were raced at an age of less than 12 months. These 107 birds were included in this analysis. Each bird was given a status of lost before races, lost during races, or race series completed. Performance measurement (marker of performance) is based on race coefficient (RC) calculated as place won divided by number of birds in the race. Hence, a lower number indicate a better performance. This is a continuous measurement of performance rather than an artificial ranking or point system. Results are reported over all races combined and by race distance. Medians are reported for each group as medians are a better reflection of group performance than means, and less susceptible to individual outlier values. Races were grouped in 3 distance categories: short distance (70–160 miles); middle distance (161–260 miles), long distance (261 miles and more).

### Double blinding

The genetic analyses were conducted before the races were flown and without the knowledge which race any particular bird was entered. The race managers did not know the outcome of genetic analyses.

Genetic analyses, including determination of gender, were conducted by the Animal Genetics Inc. 3382 Capital Cr. N.E., Tallahassee, FL 32308, www.avianbiotech.com, Toll Free + 1–800-514–9672 or + 1–850-386–1145.

Determination of mtDNA status is based on the maternal lineage in the pedigree. No genetic analyses were conducted on variations of mtDNA. Determination of color was by visual observation.

### Statistical

For the analyses of race coefficients for the effect of genotypes, the method of Kruskal–Wallis (Kruskal and Wallis (1952) was applied. Box plots were generated to display outcomes among the various factors of interest. *P* values were unadjusted for multiple testing.

## Endpoints

### Primary:

1. Comparison of median overall RC (with range) within genes *LDHA*, *DRD4*, *F-KER*, and *MSTN.*

### Secondary:

1. Comparison of median overall RC (with range) as a multivariate combining genotypes from the 4 genes *LDHA*, *DRD4*, *F-KER*, and *MSTN*
2. Comparison of median RC (with range) per distance (short, median, long) within genes *LDHA*, *DRD4*, *F-KER*, and *MSTN*
3. Comparison of median RC (with range) per distance (short, median, long) as a multivariate combining genotypes from the 4 genes *LDHA*, *DRD4*, *F-KER*, and *MSTN*
4. Comparison of median overall RC (with range) overall and per distance group for each demographic factors
5. Repeat secondary objective #3 with mtDNA added

## Results

In 2019 in one breeding loft located in the USA, a family of racing pigeons was dedicated to breeding young racing birds for participation in one loft races. All 17 breeding pairs were related to Queen L, the foundation hen for Jelle Roziers in Belgium with many high performance descendants. In these 17 pairs, the relationship with Queen L was either via the paternal line (not passing on the Queen L mtDNA) or via the maternal line (passing on the mtDNA). This homogenous family of birds is hence ideal to address this question of genetic variation as well as whether presence of Queen L mtDNA makes a difference in race performance.

The 17 pairs produced 124 birds of which 122 birds had DNA samples collected. A total of 107 birds were sent to OLRs and raced at age < 1 year old, 2 were sent to local races, 10 were sent to a yearling race (and raced at > 1 year of age), and 3 were not send to any races. Of the 107 birds, 23 carry the mtDNA of Queen L, and 84 birds did not. The data set consisted of 124 pigeons with 636 total observations. Of these, 73 birds had data from which a race coefficient could be calculated (585 observations). Of the 73 birds, 71 had mtDNA data (558 observations). Data analysis for race performance is based on the 558 observations from the 71 pigeons.

Demographics of 71 birds included in the analysis are presented in Table 1. There was no apparent difference in genetic profile between all birds bred (*n* = 122) and those with race results (*n* = 71). There were 54 checkers (76%) and 39 female (55%) birds. Given the small numbers for *MSTN* and no variance for genotype *DRD954*, no analyses were performed on these genotypes. Other genotypes had sufficient number of birds to allow statistical analyses.

**Table 1:** Characteristics of all 2019 young birds with at least 1 race record (*n* = 71)

	Race population (i.e., those with at least one race result)								
	DRD954*	DRD456*	LDHA*	F-KER*	MSTN*	Sex	mtDNA	Color	Complete/lost during
	CC	CC	AA	GG	CC	ZZ	mtDNA	B	com
*n*	71	54	0	14	67	32	17	14	39
%	100	76	0	20	94	45	24	20	55
	CT	CT	AB	TG	CT	WZ	No mtDNA	C	dur
*n*	0	16	29	29	4	39	54	54	32
%	0	23	41	41	6	55	76	76	45
	TT	TT	BB	TT				W	
*n*	0	1	42	28				3	
%	0	1	59	39				4	

Color B = blue bar, C = checker, W = white. Complete = complete the race series; During = lost during the race series

^*^Genes with their genotypes. *DRD*, dopamine receptor 4; *F-KER*, feather keratin; *LDHA*, lactate dehydrogenase A; *mtDNA*, presence of Queen L mtDNA; *No mtDNA*, absence of Queen L mtDNA

Table 2 contains the summary of race coefficients overall and the factors of interest. Race coefficients were non-normally distributed. Summaries of race coefficients include median, minimum, and maximum. Comparisons of race coefficients among levels of any factor were made using a Kruskal–Wallis test and box plots were used to display distributions.

**Table 2:** Summary statistics for race coefficients

	*N* (observations)	Med (min, max)
All	558	0.310 (0.004, 0.992)
Race length		
Short	392	0.309 (0.004, 0.992)
Middle	112	0.320 (0.009, 0.906)
Long	54	0.294 (0.012, 0.958)
Gender		
Female	267	0.300 (0.004, 0.992)
Male	291	0.318 (0.004, 0.975)
Color		
B	82	0.399 (0.012, 0.992)
C	447	0.277 (0.004, 0.980)
W	29	0.586 (0.019, 0.895)
DRD456*		
CC	423	0.335 (0.004, 0.980)
CT	125	0.205 (0.004, 0.992)
TT	10	0.286 (0.049, 0.532)
LDHA*		
AB	209	0.320 (0.007, 0.980)
BB	349	0.310 (0.004, 0.992)
F-KER*		
GG	119	0.225 (0.004, 0.975)
TG	236	0.364 (0.008, 0.992)
TT	203	0.308 (0.004, 0.958)
MSTN*		
CC	526	0.306 (0.004, 0.992)
CT	32	0.361 (0.008, 0.968)
mtDNA		
mtDNA	107	0.425 (0.012–0.992)
No mtDNA	451	0.280 (0.004–0.975)

Color B = blue, C = check, W = white

^*^Genes with their genotypes. *DRD*, dopamine receptor 4; *F-KER*, feather keratin; *LDHA*, lactate dehydrogenase A; *mtDNA*, presence of Queen L mtDNA; *No mtDNA*, absence of Queen L mtDNA

**Fig. 1:**
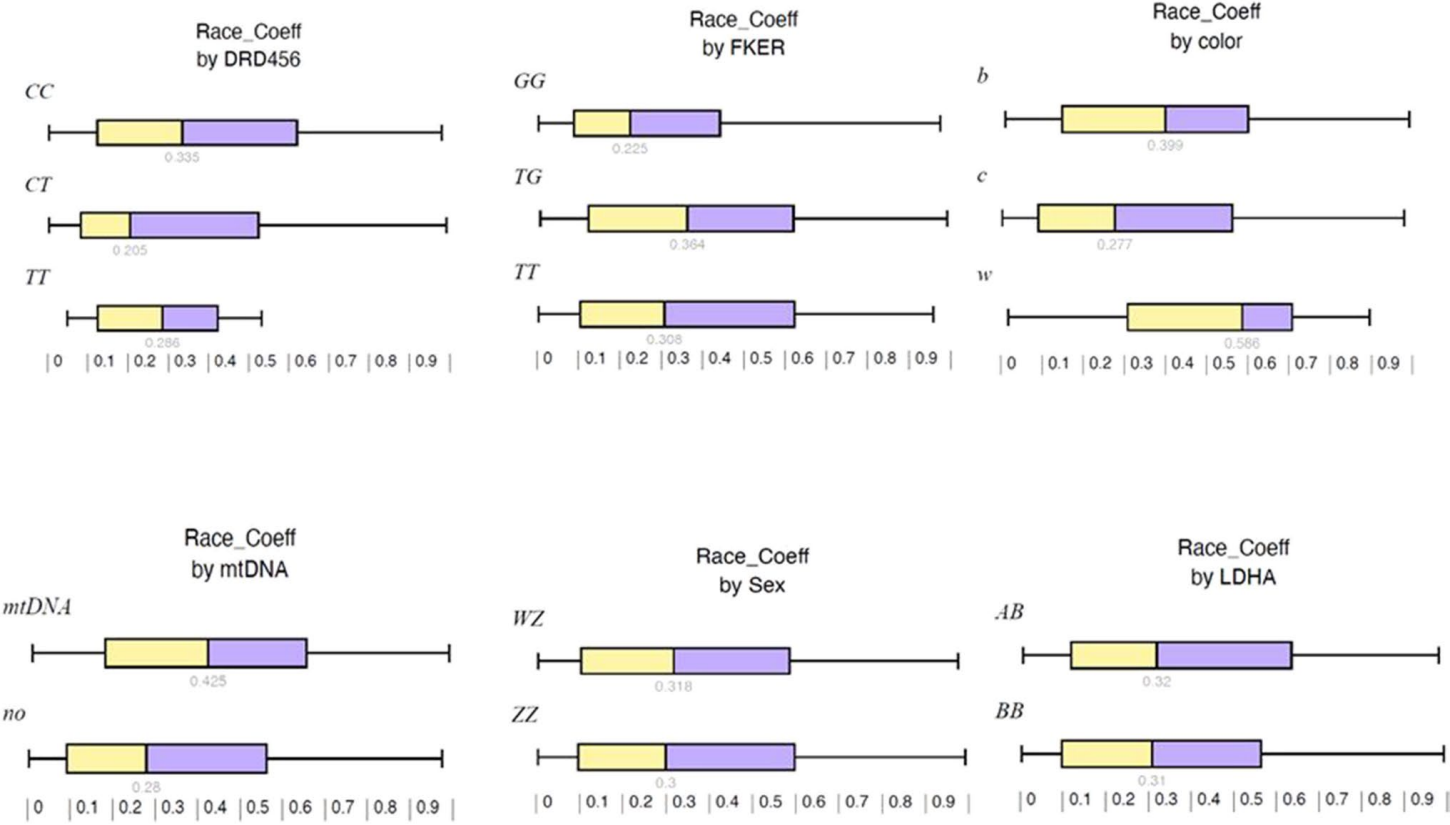
Race coefficient by individual genetic and demographic factors. Legend: DRD, dopamine receptor 4; F-KER, feather keratin; b, blue bar; c, checker; w, white; mtDNA, mitochondrial DNA of Queen L present; no, mitochondrial DNA of Queen L absent; WZ, female; ZZ, male; LDHA, lactate dehydrogenase A

For the primary analysis of race coefficients for each genes (see Fig. 1), differences were seen for *DRD456* and *F-KER* (Kruskal–Wallis *P* = 0.026 and 0.018 respectively). For *DRD456*, race coefficients were lower for *CT*. For *F-KER*, race coefficients were lower for *GG*. Exploring these genes in a multivariate model including mtDNA, both *F-KER* and *DRD456* remained significant. The effect of mtDNA alone was also significant (*P* = 0.004).

**Fig. 2:**
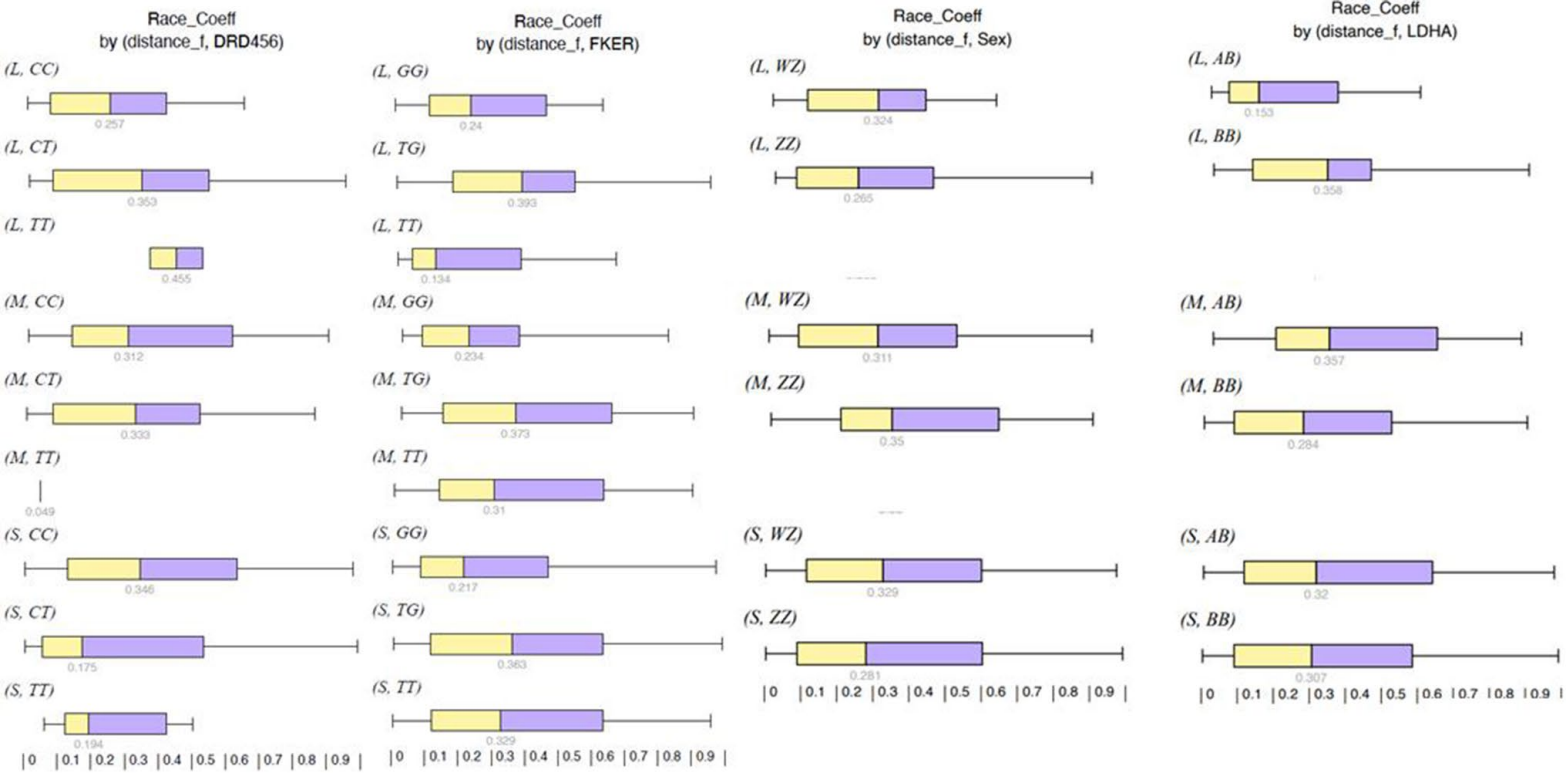
Race coefficient by distance. Legend: L, long distance; M, medium distance; S, short distance; DRD, dopamine receptor 4; F-KER, feather keratin; mtDNA, mitochondrial DNA of Queen L present; no, mitochondrial DNA of Queen L absent; WZ, female; ZZ, male; LDHA, lactate dehydrogenase A

In reviewing Fig. 2, it is remarkable that birds in the longer distances with the genotypes *F-KER TT* and/or *LDHA AB* had a low RC (0.134 and 0.153 respectively), which means that 50% of the birds with this genotype were in the top 13% or 15% of the long distance races respectively. Same was found for *DRD4 CT* in short distance races, where the race records of 50% of the birds with that genotype were in the top 17%.

**Fig. 3:**
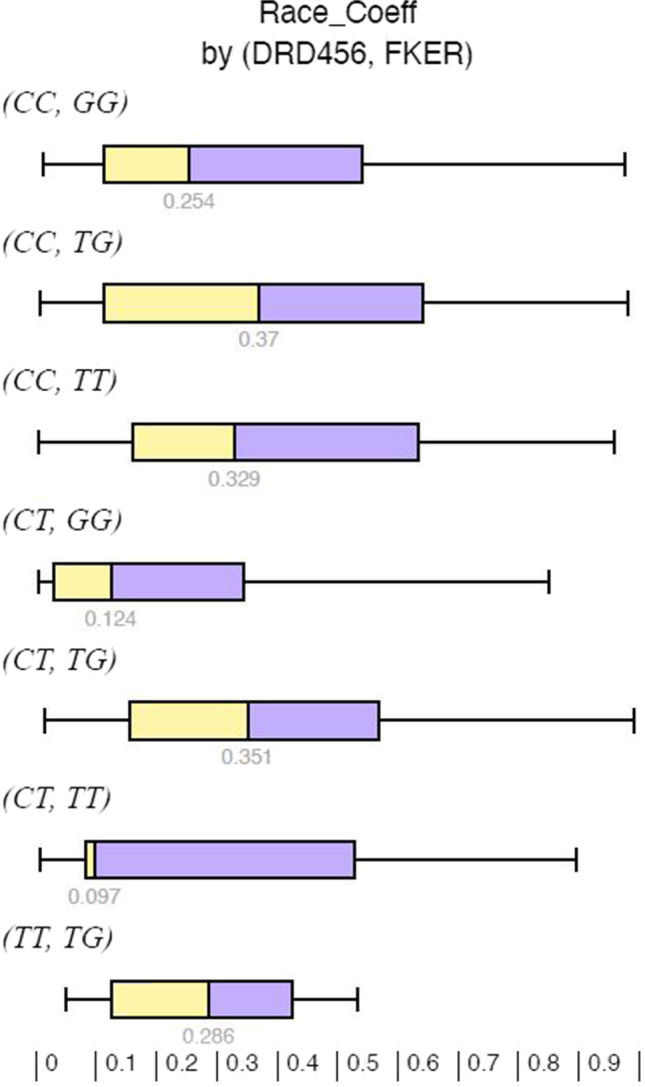
Race coefficient by *DRD4*56–*F-KER* genotype profile. Legend: DRD, dopamine receptor 4; F-KER, feather keratin

In a multivariate analysis combining genotypes of *DRD456* and *F-KER* (Fig. 3) the medians differ with *P* = 0.003. By inspection, the results suggest that genotype *DRD4*56 CT combined with *F-KER TT* had the lowest median RC. This means that in half of all the races the birds with this genotype were in the top 10%!

Effects of Queen L mtDNA on RC were previously reported by Kolvenbag and Scott (2021), where Queen L mtDNA absence seems to be related to better performance on the short distance only; there were no differences in other distance categories. Also, birds with the checker color did better overall (*P* = 0.0012), in particular on the short distance races, but also in the longer distance races. The finding needs to be interpreted with caution as only three white birds were in the dataset but contributed only 29 race coefficients. No differences in race coefficients were seen between genders.

## Discussion

In the racing population studies, there were no overall differences found in the genotypes for *LDHA* and *MSTN* (Fig. 1). For the *LDHA* genotype, it had been suggested that having an *A* as part of the genotype would result in better race performance. In this race population studied, 29/71 birds that had *AB* had a similar race performance as *BB* overall. The notion that top race and breeder pigeons have been reported to have *LDHA BB* further underlines that more studies are needed. In our prospective study, no differences were found between racing pigeons with the *LDHA* genotypes *AB* and *BB* overall, with a hint for birds with the genotype *AB* to perform better in the long distance races.

The study on variability of the *MSTN* genotype was limited by the small sample size: 32 observations from 4 birds with *CT*, while 526 observations from 67 birds had genotype *CC*; no conclusions can be drawn from our observations with regard to the *MSTN* genotype.

More interesting are the observed differences in favor of the *DRD456* genotype *CT* and the *F-KER GG* overall and *F-KER TT* in the long distance races. In a multivariate analysis combining genotypes of *DRD456* and *F-KER*, the medians significantly differ (*P* = 0.003). Racing pigeons with the genotype *DRD4*56 *CT* combined with *F-KER TT* had the lowest median RC of 0.097. This means that in half of all the races the birds with this genotype were in the top 10%!

The dopamine receptors play an essential role in daily life functions (Bhatia et al. 2021). They have been associated in humans and animals with intelligence, personality character treats, and mental capabilities, including emotions. The function of dopamine receptor 4 (*DRD4*) has been associated with cognition, impulse control, attention, and sleep (Bhatia et al. 2021). In a study by Proskura et al. (2015b) with 1380 racing records, the DRD4 genotype *CCCC* was associated with the lowest race performance. The genotypes *CTCC* and *CCCT* had better performance (statistically significant); and the *CTCT* variant very clearly had the best racing performance records. This was significant for speed and middle distance races, while on the long distance races, the performance was better also, but it was not statistically significant. In our study, we had no birds with the *CTCT* genotype, all the birds had *DRD954 CC*, and so the only variance was in *DRD456*. Our study is in line with previous reports that *DRD4 CCCT* has a better race performance than *DRD4 CCCC*. The report of Proskura et al. (2015b) does not include information on the genotype *CCTT*. In our study, the sample size of birds with that genotype was too small to draw conclusions, but seems to be closer to the *CCCC* race performance than to the race performance of birds with *DRD4 CCCT* genotype.

Variation in *F-KER* genotype may impact feather quality. While some believe that visual and tactical observation could classify good or poor feather quality; to date, only the so-called soft feather was the best possible distinguisher based on feather quality. The genetic marker *F-KER* and its variability by the loss of the cysteine at position 83 may well be an indication of differences of pigeon feather keratin that may affect the structure of feathers. By changes in feather structure, changes in biomechanical features can be expected that may influence the flying capability of racing pigeons. While our analysis indicated an overall difference in favor for the *F-KER* genotype *GG*, the race coefficient was similar over all distances. It was very interesting to see that *F-KER TT* had the best RC in the long distance races within genotype and compared to *GG* (*P* = 0.093; Kruskal–Wallis). The sample sizes in the longest races are smallest so it is expected that a larger visual separation may be a function of smaller sample sizes. However, this trend observed for the long distance races is in line with previous findings reported by Proskura et al. (2017). They reported in a study with 123 racing pigeons and 2589 race records that the *F-KER* genotype *TT* showed statistically significant (*P* ≤ 0.05) better race results than the other genotypes; however, in our study the *F-KER* genotype *GG* had the better overall performance. Also GalluVet Laboratories (n.d.) (https://www.birdsandfowl.galluvet.be/F-KER) report that the *F-KER TT* genotype variant occurs significantly more in successful pigeons on long distance flights (> 500 km) than the *F-KER GG* genotype. The fact that no numeric details are supporting these statements underlines the importance of conducting and reporting our prospective study.

Just like for other factors, there are top pigeons that possess the *G* allele, despite the negative effect on long distance performance. This suggests that one genetic marker is insufficient to identify a top performance pigeon.

Table 3 presents our study findings versus the hypotheses derived from previous reports.

**Table 3:** Hypothesis and results

Factor	Hypothesis	Study results
*LDHA*	Short distance *AA* > *AB* > *BB*	Overall no differences; trend *AB* > *BB* in longer races
*DRD4*	Longer distance *CTCT* > *CTCC* or *CCCT* > *CCCC*, *CCTT*, *TTCC*, *TTTT*	Overall *CCCT* > *CCCC* or *CCTT*, largest effect in shorter races
*F-KER*	Overall and in longer races *TT* > *TG*, or *GG*	Overall *GG* shows best result; in longer races *TT* > *GG*
*MSTN*	Longer races *TT* > *CT* > *CC*	Sample size too small to draw conclusions
mtDNA Queen L	Presence > absence in longer races	No difference in longer races; shorter races: absence > presence
Gender	Male = female	Male = female
Color	Checker = blue	Checker > blue

(> means better than)

Despite that some individual factors were statistically significant showing better performers, it is more interesting to look at more than one factor in multivariate analyses to derive at a superior performing racing pigeon. Both *DRD4* and *F-KER* were independently significant in the multivariate analysis. Looking at Fig. 3, it suggests that birds with *DRD4 CCCT* and *F-KER TT* had the lowest RC. Adding other independently significant factors like mtDNA and/or color reduced the sample size such that no meaningful conclusions could be drawn, but it is certainly peaking interest for further study.

Our results on mtDNA were reported previously (Kolvenbag and Scott 2021), where having Queen L mtDNA did not result in better race results on the long distance; actually not having Queen L mtDNA had better short distance results. In our study, checker colored birds had better race results than blue colored birds. No biological explanation is known for this; it may be related to this specific family of birds as Jelle Roziers reported similar findings in his 2020 race team that 7 out of 8 best racers were checker colored (Jelle Roziers, personal communication). It is not known if our results are specific for the family of birds studied. The maternal lineage as studied by the mtDNA relationship did not impact the effects of F-KER and DRD456 as they remained significantly different after including mtDNA in the model. We found no differences in gender; hens and cock birds had similar race coefficients. This is in line with a previous study in 2017, where also no difference in race performance was found between genders in the young bird population from the same family that year (data on file).

In conclusion, our study showed that in the population of racing pigeons studied, there was no impact on race performance by variability in gender, but that variability in genotypes for *DRD4* and *F-KER* as well as variability in mtDNA (only in short races), *LDHA* (only for long races), or/and color showed differences in race performance. While individual factors were related to differences in race performance, and although one could be tempted to favor a bird with *DRD CCCT*–*F-KER TT*–*LDHA AB*–checker color for long distance races, further and larger prospective studies including birds unrelated to our family of birds will be needed to confirm our findings and to determine a superior profile including multiple genetic factors.

## Data Availability

Data will be available upon request from the authors.
